# Antenatal Fetal Lung Volume for Predicting Neonatal Respiratory Distress Syndrome: A Systematic Review and Meta-Analysis of Diagnostic Accuracy

**DOI:** 10.3390/diagnostics16142156

**Published:** 2026-07-09

**Authors:** Kasidis Nontaprom, Potsanop Kassayanan, Monchai Suntipap

**Affiliations:** 1 Faculty of Medicine, Srinakharinwirot University, Ongkharak, Nakhon Nayok 26120, Thailand; kasidis.nontaprom@g.swu.ac.th (K.N.); pongthong.kassayanan@g.swu.ac.th (P.K.); 2 Department of Obstetrics and Gynecology, Faculty of Medicine, Srinakharinwirot University, Ongkharak, Nakhon Nayok 26120, Thailand

**Keywords:** fetal lung volume, prenatal ultrasonography, neonatal respiratory distress syndrome, diagnostic accuracy, systematic review and meta-analysis

## Abstract

**Objective**: To determine the diagnostic accuracy of antenatal ultrasonographic fetal lung volume (FLV) for predicting neonatal respiratory distress syndrome (RDS) and to identify clinically relevant sources of heterogeneity. **Methods**: A systematic review and diagnostic test accuracy meta-analysis was conducted in accordance with PRISMA-DTA and registered in PROSPERO (CRD420251150707). MEDLINE, Scopus, CINAHL Complete, and CENTRAL were searched from inception to 15 November 2025, supplemented by manual searching of reference lists and gray literature. Studies reporting antenatal ultrasonographic FLV with postnatal diagnosis of neonatal RDS were included. Diagnostic accuracy outcomes were synthesized using a bivariate random-effects model to estimate pooled sensitivity, specificity, likelihood ratios, diagnostic odds ratio (DOR), and hierarchical summary receiver operating characteristic (HSROC) curves. For studies reporting FLV as a continuous measure without prespecified cutoffs, mean differences were pooled using random-effects models. Prespecified subgroup analyses evaluated fetal population and scan-to-delivery interval. A sensitivity analysis excluding studies with non-standard RDS definitions was conducted to assess the robustness of pooled estimates. **Results**: Seven studies (n = 900) met the inclusion criteria. The pooled sensitivity and specificity of FLV for predicting neonatal RDS were 88.00% (95% CI: 78.00 to 94.00) and 82.00% (95% CI: 70.00 to 90.00), respectively, with an HSROC area under the curve of 0.93. Substantial heterogeneity was observed (I^2^ > 75% for both sensitivity and specificity). Subgroup analyses suggested lower heterogeneity in studies including mixed term and preterm fetuses and in studies assessing FLV within 24 h before delivery; however, these findings should be interpreted cautiously because of the small number of studies. In continuous outcome analyses, neonates without RDS had significantly larger FLV than those with RDS (mean difference 7.12 cm^3^, 95% CI: 5.90 to 8.34). Reporting of measurement reproducibility was limited across studies. **Conclusions**: Ultrasonographic FLV shows moderate diagnostic performance for predicting neonatal RDS and may provide adjunctive value for perinatal risk stratification, especially when measured close to delivery. However, the current evidence is limited by small study numbers, single-country data, post hoc ROC-derived thresholds, inconsistent RDS definitions, unmeasured confounding, and limited reproducibility reporting. Therefore, FLV should be regarded as an exploratory adjunctive biomarker rather than a validated stand-alone clinical test. Standardized acquisition and segmentation protocols, gestational-age-adjusted or fetal-size-adjusted thresholds, mandatory reliability metrics, and external validation are needed before routine implementation.

## 1. Introduction

Neonatal respiratory distress syndrome (RDS) remains a leading cause of early neonatal morbidity and healthcare resource utilization in neonatal care, particularly among preterm infants. The reported incidence of neonatal RDS varies across countries, ranging from 1.20% to 1.64% [1,2,3]. Neonatal RDS accounts for approximately 4.06% to 76.2% of neonatal intensive care unit (NICU) admissions [2,3,4,5], with reported mortality rates ranging from 5.1% to 55.2% [1,2,4,5,6,7]. Despite the widespread use of antenatal corticosteroids and advances in perinatal care, clinicians continue to face challenges in individualizing decisions regarding when to administer antenatal corticosteroids and preparedness for neonatal RDS management. Therefore, accurate antenatal prediction of RDS would be valuable for improving neonatal outcomes.

To date, conventional risk stratification of RDS has primarily relied on gestational age (GA), with lower GA being associated with a higher risk and more severe neonatal RDS due to surfactant insufficiency resulting from incomplete maturation of type II pneumocytes [8]. Additional risk factors, including male sex [9] and maternal gestational diabetes mellitus (GDM) [10], cesarean delivery [11], and maternal COVID-19 infection [12], have also been identified. However, these factors alone are insufficient for precise risk prediction. Biochemical tests, such as the lecithin to sphingomyelin ratio with lamellar body count in amniotic fluid, demonstrate excellent predictive performance but are infrequently used in clinical practice because of their invasiveness and limited availability [13].

Non-invasive approaches, particularly ultrasonographic imaging, may help in addressing this gap. The hypothesis that fetal lung volume (FLV) assessment is associated with fetal pulmonary growth has gained increasing interest, supported by evidence that antenatal corticosteroid administration is associated with increased FLV [14]. Although FLV primarily reflects pulmonary size and structural development rather than direct biochemical maturity, it may serve as an indirect imaging marker associated with neonatal respiratory risk. Accordingly, ultrasonographic FLV assessment has emerged as a potential tool for predicting neonatal RDS. Nevertheless, many studies evaluating ultrasonographic FLV as a potential predictor of neonatal RDS are limited by small sample sizes, heterogeneous populations, variations in measurement techniques, GA at assessment, inconsistent cutoff values, and differences in outcome definitions.

Therefore, this systematic review and meta-analysis (SRMA) aimed to synthesize available evidence on ultrasonographic FLV as a predictor of neonatal RDS and to clarify its potential role as an adjunctive antenatal imaging biomarker.

## 2. Methods

### 2.1. Study Design

This SRMA was conducted according to the Preferred Reporting Items for SRMA extension for diagnostic test accuracy (PRISMA-DTA) [15] and registered in PROSPERO under the number CRD420251150707. The completed PRISMA-DTA checklist is provided as Supplementary S1.

#### 2.1.1. Search Strategy, Screening, and Eligibility

Two reviewers independently searched electronic databases, including MEDLINE, Scopus, CINAHL Complete, and CENTRAL, supplemented by manual searching of reference lists of included studies and relevant systematic reviews via Google Scholar from inception to 15 November 2025. The search terms were developed based on the PIRD (population, index test, reference test, diagnosis of interest) framework [16]: pregnant women AND fetal lung volume AND neonatal respiratory distress syndrome with relevant search terms. The full search strategy is presented in Supplementary S2.1–S2.4. All retrieved records were exported to a reference-management file, and duplicates were removed before screening. Gray literature searches were recorded by source, search date, search terms, and number of screened records to improve reproducibility.

#### 2.1.2. Study Selection

Subsequently, two reviewers independently screened titles and abstracts using a predefined eligibility form. Full-text articles were then assessed independently by the same two reviewers. The inclusion criteria were studies involving pregnant women who underwent ultrasonographic FLV measurement and reported diagnostic performance outcomes for neonatal RDS. The exclusion criteria were studies published in non-English languages that could not be translated and studies with insufficient data for outcome pooling after three contact attempts made two weeks apart. Disagreements were documented and resolved by discussion; unresolved discrepancies were adjudicated by a third reviewer.

### 2.2. Data Extraction

Two reviewers independently extracted data using a standardized data extraction form. Extracted data included general study information, including author name, year of publication, country, study design, and sample size. Participant characteristics were recorded, including maternal age, parity, body mass index (BMI), gestational age at FLV measurement, gestational age at delivery, and antenatal corticosteroid exposure. Data on ultrasonographic FLV measurement were extracted, including cutoff values, ultrasound dimensionality, measurement technique, ultrasound equipment, and software. Scan-to-delivery interval and reproducibility metrics for FLV, including intraclass correlation coefficients for intraobserver or interobserver reliability, were extracted when available. Neonatal outcomes were collected, including the definition of neonatal RDS, number of RDS cases, birthweight, route of delivery, and method of outcome ascertainment.

Data for pooling were categorized into two types: diagnostic performance (with cutoff values) and continuous data (without cutoff values). Diagnostic performance data included sensitivity, specificity, positive and negative likelihood ratios (LR+ and LR−), diagnostic odds ratio (DOR), and the area under the receiver operating characteristic curve (AUC). For continuous data, the mean and standard deviation (SD) of FLV were extracted for neonates with and without RDS. When ultrasonographic FLV was reported as a median with range or interquartile range, these values were converted into estimated means and SDs using established methods.

When more than one FLV threshold was reported within a study, the threshold corresponding to the primary diagnostic analysis of the original study was extracted. If no primary threshold was explicitly prespecified, the ROC-derived threshold reported as the main cutoff by the original authors was used. The use of post hoc ROC-derived thresholds was incorporated into the QUADAS-2 index-test risk-of-bias assessment.

### 2.3. Risk of Bias Assessment

The quality of the included studies was independently assessed by two reviewers using the Quality Assessment of Diagnostic Accuracy Studies-2 (QUADAS-2) tool [17]. QUADAS-2 evaluates four risk-of-bias domains: patient selection, index test, reference standard, and flow and timing. It also evaluates three applicability domains: patient selection, index test, and reference standard. Judgments were categorized as low, unclear, or high risk of bias. Disagreements were resolved by consensus, with adjudication by a third reviewer when necessary.

### 2.4. Statistical Analysis

Demographic characteristics of the participants were reported using frequency data. Ultrasonographic FLV outcomes for predicting neonatal RDS were pooled into two types depending on the outcome measures. For diagnostic outcomes (with a cutoff value), the results were pooled as sensitivity, specificity, LR+, LR−, DOR, and hierarchical summary receiver operating characteristic (HSROC) curves with 95% confidence intervals (CI). Additionally, a Fagan nomogram was constructed using the pooled LR+/LR− to estimate post-test probabilities.

For continuous outcomes (without a cutoff value), the results were pooled as mean differences (MD) with 95% CI to compare FLV between neonates with and without RDS. Diagnostic accuracy outcomes were synthesized using a bivariate random-effects model, whereas continuous outcomes were pooled using a random-effects inverse-variance model. Heterogeneity among studies was assessed using the I^2^ statistic, with values exceeding 50% indicating substantial heterogeneity. In diagnostic test accuracy meta-analysis, I^2^ values for sensitivity and specificity were interpreted descriptively because apparent heterogeneity may reflect threshold effects and the correlation between sensitivity and specificity. Variation in FLV cutoff values was therefore considered a potential source of heterogeneity and was examined qualitatively across included studies. Subgroup analyses were performed to explore potential sources of heterogeneity, including fetal population and scan-to-delivery interval, when at least three studies were available. Meta-regression was planned a priori when at least ten studies were available. Publication bias was assessed using Deeks’ funnel plot asymmetry test, with *p* < 0.10 indicating statistically significant asymmetry. Because this test is underpowered when the number of studies is small, results were interpreted cautiously. All statistical analyses were performed using Stata version 18 (StataCorp LLC, College Station, TX, USA).

## 3. Results

### 3.1. Study Selection

**Figure 1:**
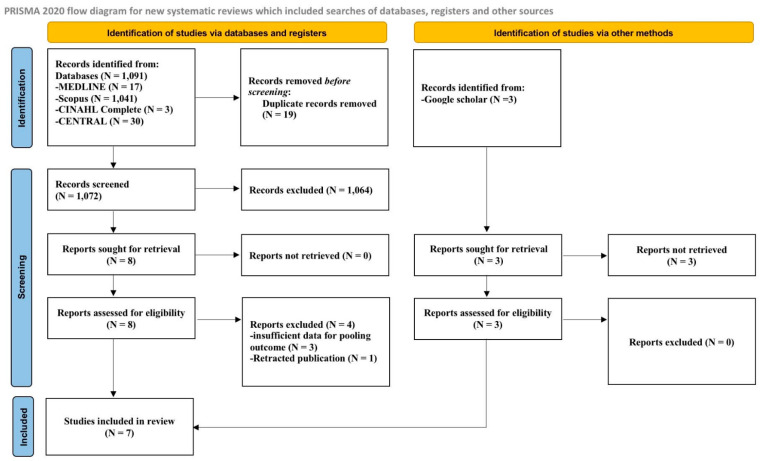
PRISMA-DTA flow diagram of study selection. The PRISMA 2020 flow diagram template was adapted from Page et al. [25], https://creativecommons.org/licenses/by/4.0/ (accessed on 4 March 2026).

A total of 1094 studies were identified from MEDLINE, Scopus, CINAHL Complete, and CENTRAL, supplemented by manual searching via Google Scholar. After removal of 19 duplicates, 1075 records remained for title/abstract screening. 1064 records were excluded as irrelevant, leaving 11 full-text articles assessed for eligibility. After full-text review, three studies were excluded due to insufficient diagnostic accuracy data, one study was excluded because the publication had been retracted, and seven studies [18,19,20,21,22,23,24] were included in the analysis, as shown in Figure 1.

#### Study Characteristics

Seven studies [18,19,20,21,22,23,24] were conducted in Egypt; six were prospective cohort studies and one was cross-sectional [22]. All studies measured ultrasonographic FLV using 3D ultrasound, with cutoff values ranging from 27.2 cm^3^ to 49.50 cm^3^. As summarized in Table 1, all included studies were small to moderate in size (n = 50–200) and reporting of reproducibility was limited.

Participant characteristics are summarized in Table 2. The fetal populations comprised term only [18], preterm only [19,22], and mixed term and preterm cohorts [20,21,23,24] GA at assessment and delivery varied across studies, and antenatal corticosteroid use was reported in two studies [21,22] Scan to delivery interval was within 24 h in five studies [18,19,20,22,23], whereas two studies did not specify this interval [21,24].

### 3.2. Diagnostic Performance of Ultrasonographic FLV to Predict Neonatal RDS

**Figure 2:**
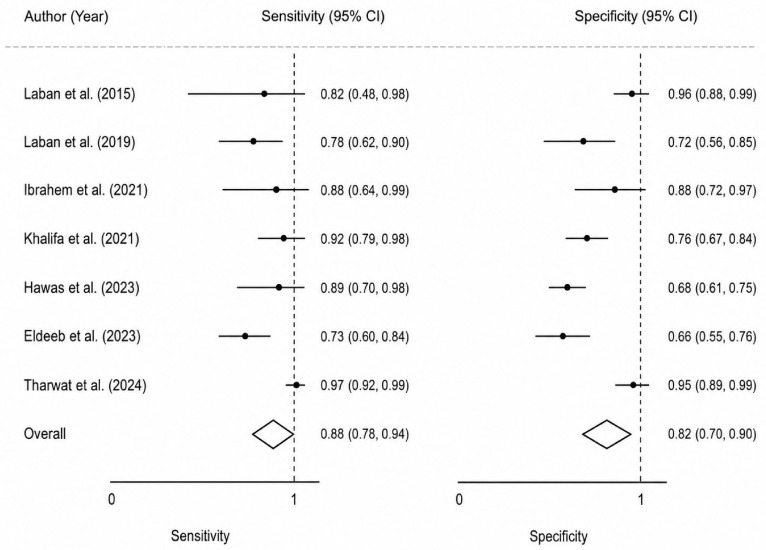
Forest plots of sensitivity and specificity of fetal lung volume for predicting neonatal respiratory distress syndrome [18,19,20,21,22,23,24].

Seven studies [18,19,20,21,22,23,24], including a total of 900 participants, evaluated ultrasonographic FLV for the prediction of neonatal RDS, of whom 318 neonates were diagnosed with RDS. The FLV cutoff values ranged from 27.20 to 49.50 cm^3^. The sensitivity of ultrasonographic FLV for predicting neonatal RDS ranged from 72.88% to 97.30%, while the specificity ranged from 65.91% to 95.70%. The LR+ ranged from 2.14 to 21.15, and the LR− ranged from 0.03 to 0.41. (Table 3). The pooled sensitivity and specificity were 88.00% (95% CI: 78.00% to 94.00%; I^2^ = 79.58%) and 82.00% (95% CI: 70.00% to 90.00%; I^2^ = 90.96%), respectively, as shown in Figure 2.

**Figure 3:**
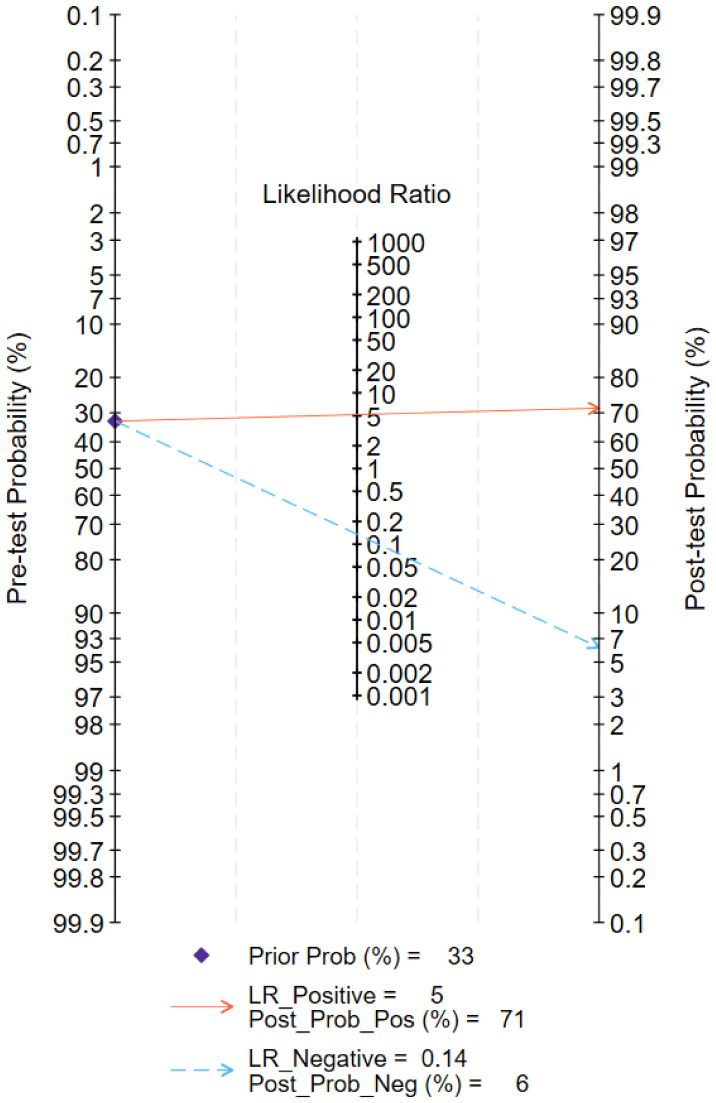
Fagan nomogram illustrating post-test probabilities of neonatal respiratory distress syndrome based on the pooled likelihood ratios of fetal lung volume, using an illustrative pre-test probability of approximately one-third.

**Figure 4:**
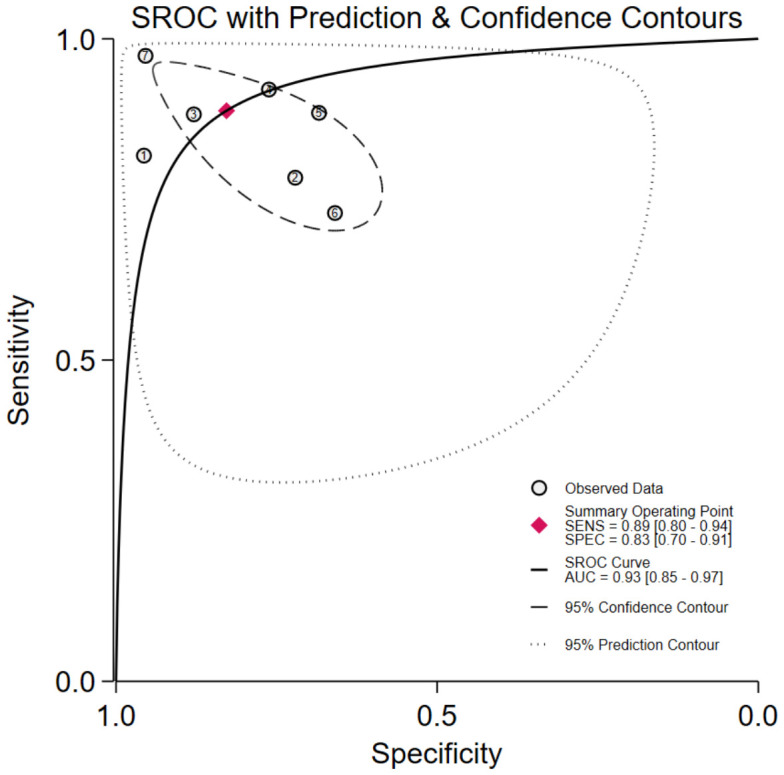
Hierarchical summary receiver operating characteristic (HSROC) curve of fetal lung volume for predicting neonatal respiratory distress syndrome.

The pooled LR+ was 5.00 (95% CI: 2.70 to 9.80; I^2^ = 86.29%), and the pooled LR− was 0.14 (95% CI: 0.07 to 0.28; I^2^ = 84.90%). The pooled DOR was 38.00 (95% CI: 10.00 to 142.00; I^2^ = 100.00%), indicating substantial imprecision and instability of the pooled estimate. Using an illustrative pre-test probability of approximately one-third, a positive test, defined as smaller FLV, increased the post-test probability to 71%, whereas a negative test, defined as larger FLV, reduced the post-test probability to 6%, as shown in the Fagan nomogram in Figure 3. The HSROC AUC was 0.93 (95% CI: 0.85 to 0.97), indicating good discrimination (Figure 4).

Subgroup analyses were restricted to two clinically relevant domains, as subgrouping by ultrasonographic technique was not feasible because all included studies measured FLV using three-dimensional ultrasonography. When stratified by GA at inclusion, studies including both term and preterm fetuses (four studies, n = 593) showed reduced heterogeneity (I^2^ = 0%), despite higher pooled sensitivity of 93.00% (95% CI: 86.00% to 97.00%) and specificity of 84.00% (95% CI: 69.00% to 93.00%). This subgroup yielded a pooled LR+ of 5.80 (95% CI: 2.70 to 12.60) and LR− of 0.08 (95% CI: 0.03 to 0.19), corresponding to a DOR of 74.00 (95% CI: 16.00 to 337.00) and an AUC of 0.96 (95% CI: 0.91 to 0.98). However, subgroup analyses among studies including only preterm fetuses were not performed because too few studies were available to allow meaningful comparisons. These findings should be interpreted cautiously because this subgroup included only four studies and may have combined populations with substantially different baseline RDS risks and clinical indications for testing.

Subgroup analysis according to the timing of FLV measurement relative to delivery partially characterized sources of heterogeneity. Studies in which FLV was assessed within 24 h prior to delivery (five studies, n = 650) demonstrated no detectable statistical heterogeneity within this subgroup (I^2^ = 0%). The pooled sensitivity and specificity in this subgroup were 83.00% (95% CI: 74.00% to 90.00%) and 77.00% (95% CI: 64.00% to 87.00%), respectively. The pooled LR+ was 3.70 (95% CI: 2.10 to 6.40) and LR− was 0.22 (95% CI: 0.13 to 0.37), yielding a DOR of 17.00 (95% CI: 6.00 to 45.00) and an AUC of 0.87 (95% CI: 0.84 to 0.90). However, subgroup analyses for time-unspecified fetal lung volume (FLV) were not performed because only two studies were included. The absence of detectable statistical heterogeneity in this subgroup should not be interpreted as proof of true homogeneity because the number of included studies was limited.

#### 3.2.1. Comparison of the Mean Ultrasonographic FLV Between Neonates with and Without RDS

**Figure 5:**
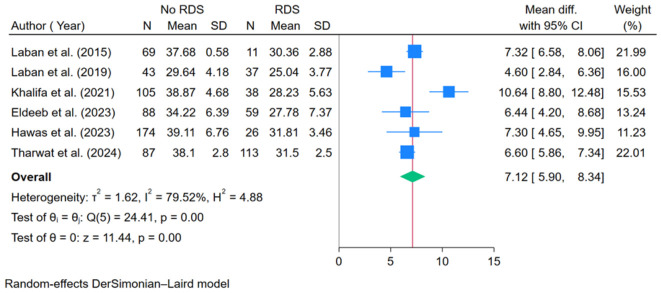
Forest plot of mean differences in fetal lung volume between neonates without and with respiratory distress syndrome [18,19,20,22,23,24]. Blue squares represent study-specific mean differences, with square size proportional to study weight. Horizontal lines indicate 95% confidence intervals. The green diamond represents the pooled mean difference.

Six studies [18,19,20,22,23,24], including 850 participants, reported the mean and SD of ultrasonographic FLV in neonates with and without RDS (Table 4). Neonates without RDS had a significantly larger FLV than those with RDS, with a pooled MD of 7.12 cm^3^ (95% CI: 5.90 to 8.34; *p* < 0.001). However, substantial heterogeneity was observed (I^2^ = 79.52%), as shown in Figure 5.

#### 3.2.2. Risk of Bias Assessment

Methodological quality was assessed using the QUADAS-2 tool. Overall, all included studies were judged to have a high risk of bias, driven primarily by a high risk in the index-test domain, mainly due to the use of non-prespecified, ROC-derived fetal lung volume cut-off values. Patient selection and reference-standard domains were rated as low to unclear risk across studies. Applicability concerns were generally low, except for the reference standard in two studies that used broader respiratory distress composite outcomes rather than isolated neonatal RDS. Inconsistent reporting of neonatal RDS definitions may have introduced reference-standard misclassification bias because postnatal respiratory distress is not synonymous with surfactant-deficiency RDS. Importantly, this pattern reflects methodological limitations intrinsic to early exploratory diagnostic studies—particularly those focused on threshold derivation—rather than systematic flaws in study conduct. The inconsistency in RDS reference standards across studies, with two studies employing composite respiratory distress outcomes rather than isolated neonatal RDS, represents an additional source of applicability concern that may affect the interpretation of pooled diagnostic accuracy, as summarized in Table 5.

### 3.3. Publication Bias

Deeks’ funnel plot asymmetry test detected no statistically significant asymmetry (*p* = 0.50). However, because only seven studies were included, this test was underpowered, and the absence of statistically significant asymmetry should not be interpreted as evidence against publication bias. (Supplementary S3).

## 4. Discussion

This SRMA evaluated the diagnostic performance of ultrasonographic FLV for predicting neonatal RDS across GA groups. Across 900 pregnancies in seven studies, FLV demonstrated moderate diagnostic performance, although substantial heterogeneity was observed, indicating important between-study differences in populations and index test implementation. Therefore, the pooled diagnostic accuracy estimates should be interpreted cautiously and should not be considered evidence that FLV is a validated stand-alone diagnostic test.

Subgroup analyses suggested higher diagnostic performance among studies including both term and preterm pregnancies. However, this finding should be interpreted cautiously because these cohorts may differ substantially in baseline RDS risk, clinical indication for testing, and delivery context. Although no detectable statistical heterogeneity was observed in this subgroup, the small number of studies limits the ability to infer true homogeneity.

The timing of FLV assessment appeared to be an important factor associated with study consistency. Studies in which FLV was measured within 24 h before delivery demonstrated no detectable statistical heterogeneity within that subgroup (I^2^ = 0%), whereas substantial heterogeneity remained among studies without a specified scan-to-delivery interval (I^2^ = 79.10%), indicating that subgroup analysis did not fully account for the sources of heterogeneity across the entire evidence base. Because fetal pulmonary growth and respiratory risk evolve across gestation, FLV assessed closer to delivery may be more closely associated with respiratory status at birth and the risk of clinically relevant RDS at the time of neonatal transition. Accordingly, pooled cutoffs derived from mixed populations and heterogeneous timing should be interpreted cautiously, particularly when extrapolated across GA groups with markedly different baseline risks.

For continuous outcomes, neonates who developed RDS had significantly smaller FLV than those without RDS, supporting FLV as a marker of fetal pulmonary growth that may be associated with neonatal respiratory outcomes. This association should not be interpreted as evidence that FLV directly measures pulmonary maturity or independently predicts neonatal RDS because adjusted and longitudinal evidence remains insufficient. This finding is consistent with previous studies demonstrating that antenatal corticosteroid exposure is associated with increased FLV [14], which may indirectly contribute to a reduced risk of neonatal RDS. Nevertheless, observational associations between FLV and RDS do not imply that FLV alone can define clinical maturity, especially in the presence of protocol variability and threshold inconsistency. A further consideration is the potential influence of uncontrolled confounders on pooled estimates. Antenatal corticosteroid administration, gestational age at delivery, birthweight, fetal growth restriction, maternal diabetes, elective cesarean delivery, multiple pregnancy, fetal sex, and indication for delivery all influence neonatal RDS risk. However, none of the included studies performed adjusted analyses, and individual participant data were not available to generate pooled adjusted estimates. Smaller FLV may reflect earlier gestational age or smaller fetal size rather than independent pulmonary maturity. Antenatal corticosteroid use was reported in only two included studies, precluding a formal subgroup analysis by corticosteroid exposure. Because corticosteroids may influence both FLV and neonatal RDS risk, differential corticosteroid exposure across studies may have contributed to heterogeneity in both FLV measurements and outcome rates.

Other ultrasonographic markers, including main pulmonary artery Doppler indices [20,24,26,27,28,29,30,31,32,33] and the fetal lung-to-liver intensity ratio [20,24,34], have also been evaluated for antenatal prediction of neonatal RDS. However, their reported diagnostic performance and cutoff values vary substantially across studies, limiting immediate clinical applicability. These inconsistencies reinforce the need for standardized acquisition methods, prespecified thresholds, and external validation before any imaging marker can be implemented in routine clinical practice.

From a clinical perspective, ultrasonographic FLV may provide additional information for perinatal risk stratification in pregnancies at increased risk of neonatal RDS, particularly when delivery is anticipated within 24 h. However, FLV should be interpreted together with GA, antenatal corticosteroid exposure, and other clinical findings, rather than being used as a stand-alone diagnostic test. Diagnostic discrimination should be distinguished from clinical utility. Even if FLV shows good discrimination in pooled analysis, this does not establish that FLV improves clinical decision-making beyond established predictors such as gestational age, antenatal corticosteroid exposure, delivery indication, fetal growth, maternal diabetes, and mode of delivery. Future studies should assess incremental predictive value, calibration, decision-curve analysis, and comparison with established clinical or multivariable prediction models.

The Fagan nomogram should be interpreted cautiously because the pre-test probability was derived from the included studies and may not represent real-world populations. Baseline RDS risk differs substantially among low-risk term elective cesarean deliveries, late preterm pregnancies, very preterm pregnancies, corticosteroid-exposed pregnancies, pregnancies with diabetes, and pregnancies complicated by fetal growth restriction. Therefore, post-test probabilities based on pooled prevalence may not be directly transferable to local clinical settings.

The strengths of this SRMA include adherence to the PRISMA-DTA guidelines, providing a standardized framework for the evaluation of diagnostic accuracy. Subgroup analyses were performed based on fetal population and scan-to-delivery interval to characterize sources of heterogeneity and to reflect outcomes specific to each population. Nevertheless, several important limitations must be acknowledged. First, all included studies were conducted in Egypt, creating a single-country evidence base that limits generalizability to other ultrasound platforms, operator training environments, neonatal care pathways, obstetric management systems, and populations with different baseline risks of RDS. External validation in geographically and clinically diverse cohorts is therefore essential before FLV thresholds can be adopted in routine practice. Second, subgroup analyses did not fully account for all sources of heterogeneity. Although subgroup analyses were conducted to explore potential sources of heterogeneity, meta-regression could not be performed because fewer than ten studies were available. More than half of the included studies enrolled mixed term and preterm populations, which may not accurately represent the population at highest risk for neonatal RDS. Furthermore, post hoc ROC-derived thresholds optimized within individual datasets may inflate sensitivity, specificity, likelihood ratios, diagnostic odds ratios, and HSROC AUC. Threshold effects are therefore likely to have contributed to the observed heterogeneity and limit the interpretability of pooled sensitivity and specificity. Third, all included studies were rated as high risk of bias in the index-test domain of QUADAS-2 because cutoff values were derived post hoc from ROC analyses without predefined thresholds. This approach may overestimate diagnostic performance by optimizing thresholds within individual datasets, while the resulting threshold heterogeneity limits the reliability of the pooled estimates and precludes the establishment of a universally applicable FLV cutoff for clinical practice. Fourth, important potential confounders were not controlled for, including antenatal corticosteroid use, gestational age at delivery, birthweight, fetal growth restriction, maternal diabetes, elective cesarean delivery, multiple pregnancy, fetal sex, and indication for delivery, all of which independently influence the risk of neonatal RDS. Fifth, the diagnostic criteria for neonatal RDS were inconsistent across the included studies, and several studies did not explicitly report the definition of RDS. Respiratory distress after birth is not synonymous with surfactant-deficiency RDS. Transient tachypnea of the newborn, pneumonia, infection, meconium aspiration, pulmonary hypertension, and delayed neonatal transition may overlap clinically. Therefore, inconsistent outcome definitions may have introduced reference-standard misclassification bias and affected the pooled diagnostic accuracy estimates. Sixth, the pooled MD in FLV should be interpreted cautiously because absolute FLV values are influenced by GA and fetal size, which may limit the clinical interpretability of volume differences across heterogeneous study populations. In addition, measurement reproducibility was poorly reported across the included studies. The absence of robust intraobserver and interobserver reliability data represents a major barrier to clinical implementation because FLV is an operator-dependent imaging biomarker. Future diagnostic accuracy studies should mandatorily report reproducibility metrics, including intraclass correlation coefficients for repeated FLV acquisition and segmentation.

Future research should prioritize well-designed multicenter prospective studies with standardized FLV acquisition and segmentation protocols. These studies should evaluate gestational-age-specific centiles, observed-to-expected FLV, and fetal-size-adjusted indices rather than relying solely on absolute volume thresholds. Prospective studies should also assess whether FLV adds incremental predictive value beyond established clinical predictors, particularly in clinically relevant risk groups such as late preterm pregnancies, elective cesarean deliveries, pregnancies after antenatal corticosteroid administration, maternal diabetes, and fetal growth restriction.

## 5. Conclusions

Ultrasonographic FLV may offer adjunctive value in predicting neonatal RDS across term and preterm populations. However, the current evidence is limited by small study numbers, single-country data, post hoc ROC-derived thresholds, inconsistent RDS definitions, unmeasured confounding, and limited reproducibility reporting. Therefore, FLV should be regarded as an exploratory adjunctive biomarker rather than a validated stand-alone clinical test. The most consistent performance was observed when FLV was assessed within 24 h before delivery, suggesting that timing and protocol standardization are critical to clinical translation. Further well-designed multicenter prospective studies using standardized image acquisition and FLV segmentation protocols, gestational-age-adjusted or fetal-size-adjusted thresholds, mandatory reporting of measurement reproducibility, external validation, and prospective evaluation in clinically relevant risk groups are needed to define the clinical utility of FLV.

## Figures and Tables

**Table 1:** General characteristics of included studies.

Study (Year)	Country	Design	Sample Size (Neonate with No RDS vs. with RDS)	Ultrasound Type and Software	Cut-Off (cm^3^)	RDS Definition	True Positive	False Positive	False Negative	True Negative
Laban et al. (2015) [18]	Egypt	Prospective	80 (69 vs. 11)	3D, Voluson E6	32	NR	9	3	2	66
Laban et al. (2019) [19]	Egypt	Prospective	80 (43 vs. 37)	3D, Voluson E6	27.2	PaO_2_ < 50 or central cyanosis in room air/O_2_ needed to maintain PaO_2_ > 50 + typical CXR.	29	12	8	31
Khalifa et al. (2021) [20]	Egypt	Prospective	143 (105 vs. 38)	3D, Medison SonoAce X6	35.75	Clinical RD and/or neonatal lung US consistent with RDS (consolidation with air bronchograms; bilateral white lung).	35	25	3	80
Ibrahem et al. (2021) [21]	Egypt	Prospective	50 (16 vs. 34)	3D, Voluson E6	49.5	NR	15	4	2	29
Eldeeb et al. (2023) [22]	Egypt	Cross-sectional	147 (88 vs. 59)	3D, Voluson E6	28	NR	43	30	16	58
Hawas et al. (2023) [23]	Egypt	Prospective	200 (174 vs. 26)	3D, Samsung medison H6	35	NR	23	55	3	119
Tharwat et al. (2024) [24]	Egypt	Prospective	200 (87 vs. 113)	3D, Voluson E6	34.94	NR	110	4	3	83

Note: Neonatal RDS definitions varied across studies and were based on postnatal clinical criteria as reported in each original study. NR indicates that the original study did not explicitly report diagnostic criteria for neonatal RDS. Abbreviations: RDS = respiratory distress syndrome; RD = respiratory distress; CXR = chest X-ray; PaO_2_ = arterial oxygen tension; FiO_2_ = fraction of inspired oxygen; 2D = two-dimensional ultrasound; 3D = three-dimensional ultrasound; NR = not reported.

**Table 2:** Demographic characteristics of participants.

Study (Year)	N	Population (GA)	Mean Age (Years)	BMI (kg/m^2^)	Nulliparous (%)	Mean GA at FLV Measure (Weeks)	Mean GA at Delivery (Weeks)	Steroid n (%)	CD n (%)	Mean BBW (g)	Scan-to-Delivery Interval (Hours)
Laban et al. (2015) [18]	80	Term (37–40.wks)	NR	NR	NR	NR	38.67	NR	24 (29.66)	NR	24
Laban et al. (2019) [19]	80	Preterm (32–36.wks)	28.56	NR	NR	33.56	33.56	NR	35 (43.7)	2645	24
Khalifa et al. (2021) [20]	143	Preterm to term (32–40.wks)	29.50	26.6	NR	NR	36.5	50 (34.97)	78 (54.5)	2757.48	24
Ibrahem et al. (2021) [21]	50	Late preterm to term (34–40.wks)	23.90	NR	NR	NR	NR	12 (24)	27 (54)	NR	NR
Eldeeb et al. (2023) [22]	147	Preterm (32–36.wks)	29.87	NR	NR	NR	33.79	NR	73 (49.66)	2213.33	24
Hawas et al. (2023) [23]	200	Late preterm to term (36–40.wks)	26.9	NR	NR	37.84	NR	NR	116 (58)	3395	24
Tharwat et al. (2024) [24]	200	Preterm to term (32–39.wks)	28.80	NR	14 (7)	35.1	NR	NR	149 (74.5)	NR	NR

Note: GA = gestational age; BMI = body mass index; CD = cesarean delivery; BBW = birth body weight; NR = not reported.

**Table 3:** Data from studies reporting diagnostic performance of ultrasonographic fetal lung volume to predict neonatal respiratory distress syndrome.

Study (Year)	Cut-Off Value (cm^3^)	Sensitivity (%)	Specificity (%)	Positive Predictive Value (%)	Negative Predictive Value (%)	Likelihood Ratio Positive	Likelihood Ratio Negative
Laban et al. (2015) [18]	32	81.80	95.70	75.00	97.10	19.02	0.19
Laban et al. (2019) [19]	27.2	79.17	73.08	70.70	79.50	2.94	0.29
Khalifa et al. (2021) [20]	35.75	92.10	76.20	58.30	96.40	3.87	0.10
Ibrahem et al. (2021) [21]	49.5	87.80	88.20	93.80	77.80	7.44	0.14
Eldeeb et al. (2023) [22]	28	72.88	65.91	58.90	78.40	2.14	0.41
Hawas et al. (2023) [23]	35	88.50	68.40	29.50	97.50	2.80	0.17
Tharwat et al. (2024) [24]	34.94	97.30	95.40	96.49	96.51	21.15	0.03

Note: Sensitivity and specificity were extracted as reported in each original study. PPV, NPV, and likelihood ratios were calculated based on the reconstructed 2 × 2 tables (TP, FP, FN, TN) using the study sample sizes and reported sensitivity/specificity; minor discrepancies may occur due to rounding in the original reports.

**Table 4:** Mean and standard deviation of fetal lung volume in neonates with and without respiratory distress syndrome.

Study (Year)	Neonates Without RDS, n	FLV in Neonates Without RDS, Mean ± SD, cm^3^	Neonates with RDS, n	FLV in Neonates with RDS, Mean ± SD, cm^3^
Laban et al. (2015) [18]	69	37.68 ± 0.58	11	30.36 ± 2.88
Laban et al. (2019) [19]	43	29.64 ± 4.18	37	25.04 ± 3.77
Khalifa et al. (2021) [20]	105	38.87 ± 4.68	38	28.23 ± 5.63
Eldeeb et al. (2023) [22]	88	34.22 ± 6.39	59	27.78 ± 7.37
Hawas et al. (2023) [23]	174	39.11 ± 6.76	26	31.81 ± 3.46
Tharwat et al. (2024) [24]	87	38.1 ± 2.8	113	31.50 ± 2.5

Note: SD = standard deviation; RDS = respiratory distress syndrome.

**Table 5:** Risk of bias of included studies assessed using Quality Assessment of Diagnostic Accuracy Studies-2.

Author (Year)	Risk of Bias				Applicability Concerns			Overall
	Patient Selection	Index Test	Reference Standard	Flow and Timing	Patient Selection	Index Test	Reference Standard	
Laban et al. (2015) [18]	Unclear	High	Unclear	Low	Low	Low	Low	High
Laban et al. (2019) [19]	Low	High	Low	Low	Low	Low	Low	High
Khalifa et al. (2021) [20]	Low	High	Unclear	Low	Low	Low	Low	High
Ibrahem et al. (2021) [21]	Unclear	High	Unclear	Unclear	Low	Low	Unclear	High
Eldeeb et al. (2023) [22]	Unclear	High	Low	Unclear	Low	Low	Low	High
Hawas et al. (2023) [23]	Low	High	Unclear	Low	Low	Low	Low	High
Tharwat et al. (2024) [24]	Unclear	High	Unclear	Unclear	Low	Low	Unclear	High

Note: High = high risk of bias; Unclear = unclear risk of bias; Low = low risk of bias.

## Data Availability

All data used in this meta-analysis were extracted from published studies available in the public domain. The dataset generated during the current study, including extracted variables and statistical code, is available from the corresponding author upon reasonable request. No individual participant data were collected.

## Supplementary Materials

The following supporting information can be downloaded at: https://www.mdpi.com/article/10.3390/diagnostics16142156/s1, Supplementary S1: PRISMA-DTA checklist; Supplementary S2.1: Search strategy in MEDLINE; Supplementary S2.2: Search strategy in Scopus; Supplementary S2.3: Search strategy in CINAHL Complete; Supplementary S2.4: Search strategy in CENTRAL; Supplementary S3: Funnel plot asymmetry test for publication bias in diagnostic accuracy studies of fetal lung volume.
