# Supplementary material for: Antenatal Fetal Lung Volume for Predicting Neonatal Respiratory Distress Syndrome: A Systematic Review and Meta-Analysis of Diagnostic Accuracy
Source: Diagnostics (Basel). 2026 Jul 9;16(14):2156. doi: 10.3390/diagnostics16142156 (PMC13409002; doi:10.3390/diagnostics16142156)
Supplement: Supplementary file 1 [file diagnostics-16-02156-s001.zip › diagnostics-4372094-supplementary.pdf]

Supplementary

Supplementary S1: PRISMA-DTA checklist

Item No.	Item	Page No.
TITLE		
1	Identify the report as a systematic review and/or meta-analysis of diagnostic test accuracy.	1
ABSTRACT		
2	Structured summary including: background, objectives, data sources, study eligibility criteria, participants, index test(s), reference standard, results (number of studies, participants, estimates of diagnostic accuracy), and conclusions.	1
INTRODUCTION		
3	Scientific and clinical background, including the intended use and clinical role of the index test.	2-3
4	Objectives: to identify the review question in terms of participants, index test(s), reference standard, and diagnoses of interest.	3
METHODS		
5	Protocol and registration: indicate whether a review protocol exists and if so, provide registration information including registration number.	3
6	Eligibility criteria: inclusion and exclusion criteria for studies, and how they were applied. Specified inclusion of only published data, only certain study designs, certain languages, or only data from specific countries.	4
7	Information sources: databases searched, starting date of searches, and date last searched.	3-4
8	Search strategy: full electronic search strategy for at least one database including terms used, so it could be repeated.	Supplementary S2.1-S2.4
9	Study selection: process of study selection, including who screened titles/abstracts, who assessed full-text articles for eligibility, how many reviewers performed each process, and whether they worked independently.	4
10	Data extraction: process of data extraction, including who extracted data, how many reviewers performed the extraction, and whether they worked independently.	4

Item No.	Item	Page No.
11	Data items: list all variables for which data were sought including QUADAS-2 elements, and any assumptions or simplifications.	4-5
12	Risk of bias: method of assessing risk of bias of individual studies and concerns regarding applicability, including which tool(s) were used and how many reviewers performed the assessment.	5
13	Statistical analysis and synthesis: main summary measures (e.g. sensitivity and specificity, likelihood ratios, diagnostic odds ratio); method(s) used for quantitative synthesis; any additional analyses performed (subgroup analyses, sensitivity analyses, meta-regression).	5-6
RESULTS		
14	Study selection: number of studies screened, assessed for eligibility, and included in the review, with reasons for exclusions at each stage, ideally with a flow diagram.	6, Figure 1
15	Study characteristics: for each study, present characteristics of study participants, the index test(s), reference standard, study design, and time period of the study.	6-7, Tables 1-2
16	Risk of bias: present risk of bias assessments and concerns regarding applicability for each included study.	10, Table 5
17	Index test results: for each included study, report the number of participants, the number with the target condition, and results of the index test.	7, Tables 1, 3
18	Estimates of diagnostic accuracy: present estimates of diagnostic accuracy and measures of statistical uncertainty (e.g. 95% CI). In meta-analyses, present a summary estimate of diagnostic accuracy with measures of statistical uncertainty.	7-9, Figures 2-4
19	Heterogeneity: results of any investigation of heterogeneity including subgroup analyses and meta-regression.	8-9
20	Additional analyses: results of any additional analyses performed (e.g. sensitivity analyses).	9
DISCUSSION		
21	Summary of evidence: summarize the main findings and discuss their clinical applicability.	10-12
22	Limitations: discuss limitations at the study and review level (e.g. spectrum bias, incorporation bias, partial or	11-13

Item No.	Item	Page No.
	differential verification, and imprecise reference standard).	
23	Conclusions: provide a general interpretation of the results in the context of other evidence and implications for future research.	13
FUNDING		
24	Describe sources of funding and other support; role of funders.	13

Reference: McInnes MDF, Moher D, Thombs BD, et al. Preferred Reporting Items for a Systematic Review and Meta-analysis of Diagnostic Test Accuracy Studies: The PRISMA-DTA Statement. JAMA. 2018;319(4):388-396.

Supplementary S2.1: Search strategy in MEDLINE

Domain	Subdomain	Query	Search term	Number
Population (P)	P1 pregnant women	#1	Pregnancy	1,223,661
		#2	Pregnant women	178,326
		#3	Pregnant people	30,491
		#4	#1 OR #2 OR #3	1,242,110
	P2 Neonates	#5	Preterm	116,226
		#6	Preterm birth	97,804
		#7	Premature birth	75,800
		#8	Preterm fetus	11,650
		#9	Preterm labor	46,993
		#10	Low gestational age	38,758
		#11	Term	1,728,966
		#12	Term birth	64,557
		#13	Term fetus	28,597
		#14	Term labor	180,882
		#15	Newborn	907,543
		#16	Neonate	923,048
		#17	#5 OR #6 OR #7 OR #8 OR #9 OR #10 OR #11 OR #12 OR #13 OR #14 OR #15 OR #16	2,697,655
Total P	P1+P2	#18	#4 AND #17	390,245
Index test (I)	I1 Fetal lung volume	#19	Fetal lung volume	1,914
		#20	Lung volume measurements	48,141
		#21	#19 OR #20	49,342
	I2 ultrasound	#22	Ultrasound	2,268,086
Total I	I1 + I2	#23	#21 AND #22	5,528
Reference test (R)				
Diagnosis of interest (D)	D1 Respiratory distress syndrome	#24	"Respiratory distress syndrome"	63,061
Total		#25	#18 AND #23 AND #24	17

Supplementary S2.2: Search strategy in Scopus

Domain	Subdomain	Query	Search term	Number
Population (P)	P1 pregnant women	#1	ALL(Pregnancy)	2,172,991
		#2	ALL(Pregnant women)	638,373
		#3	ALL(Pregnant people)	123,896
		#4	#1 OR #2 OR #3	2,348,231
	P2 Neonates	#5	ALL(Preterm)	449,923
		#6	ALL(Preterm birth)	309,057
		#7	ALL(Premature birth)	239,469
		#8	ALL(Preterm fetus)	105,041
		#9	ALL(Preterm labor)	99,201
		#10	ALL(Low gestational age)	220,974
		#11	ALL(Term)	15,847,060
		#12	ALL(Term birth)	696,693
		#13	ALL(Term fetus)	222,546
		#14	ALL(Term labor)	506,120
		#15	ALL(Newborn)	1,589,809
		#16	ALL(Neonate)	549,713
		#17	#5 OR #6 OR #7 OR #8 OR #9 OR #10 OR #11 OR #12 OR #13 OR #14 OR #15 OR #16	17,214,166
Total P	P1+P2	#18	#4 AND #17	1,069,373
Index test (I)	I1 Fetal lung volume	#19	ALL(Fetal lung volume)	51,612
		#20	ALL(Lung volume measurements)	242,673
		#21	#19 OR #20	275,510
	I2 ultrasound	#22	ALL(Ultrasound)	1,989,951
Total I	I1 + I2	#23	#21 AND #22	36,404
Reference test (R)				
Diagnosis of interest (D)	D1 Respiratory distress syndrome	#24	ALL("Respiratory distress syndrome")	250,017
Total		#25	#18 AND #23 AND #24	1,041

Supplementary S2.3: Search strategy in CINAHL Complete

Domain	Subdomain	Query	Search term	Number
Population (P)	P1 pregnant women	#1	Pregnancy	292,017
		#2	Pregnant women	61,765
		#3	Pregnant people	2,745
		#4	#1 OR #2 OR #3	303,757
	P2 Neonates	#5	Preterm	45,604
		#6	Preterm birth	40,370
		#7	Premature birth	38,330
		#8	Preterm fetus	2,539
		#9	Preterm labor	5,579
		#10	Low gestational age	12,270
		#11	Term	511,081
		#12	Term birth	22,154
		#13	Term fetus	4,642
		#14	Term labor	9,289
		#15	Newborn	172,999
		#16	Neonate	32,227
		#17	#5 OR #6 OR #7 OR #8 OR #9 OR #10 OR #11 OR #12 OR #13 OR #14 OR #15 OR #16	694,496
Total P	P1+P2	#18	#4 AND #17	82,358
Index test (I)	I1 Fetal lung volume	#19	Fetal lung volume	319
		#20	Lung volume measurements	4,249
		#21	#19 OR #20	4,444
	I2 ultrasound	#22	Ultrasound	98,184
Total I	I1 + I2	#23	#21 AND #22	154
Reference test (R)				
Diagnosis of interest (D)	D1 Respiratory distress syndrome	#24	"Respiratory distress syndrome"	18,720
Total		#25	#18 AND #23 AND #24	3

Supplementary S2.4: Search strategy in CENTRAL

Domain	Subdomain	Query	Search term	Number
Population (P)	P1 pregnant women	#1	Pregnancy	89,592
		#2	Pregnant women	29,559

		#3	Pregnant people	6,456
		#4	#1 OR #2 OR #3	96,691
	P2 Neonates	#5	Preterm	20,297
		#6	Preterm birth	12,128
		#7	Premature birth	13,166
		#8	Preterm fetus	1,704
		#9	Preterm labor	3,653
		#10	Low gestational age	8,056
		#11	Term	303,458
		#12	Term birth	14,792
		#13	Term fetus	3,101
		#14	Term labor	6,628
		#15	Newborn	39,891
		#16	Neonate	43,736
		#17	#5 OR #6 OR #7 OR #8 OR #9 OR #10 OR #11 OR #12 OR #13 OR #14 OR #15 OR #16	272,155
Total P	P1+P2	#18	#4 AND #17	30,792
Index test (I)	I1 Fetal lung volume	#19	Fetal lung volume	684
		#20	Lung volume measurements	24,537
		#21	#19 OR #20	24,886
	I2 ultrasound	#22	Ultrasound	58,426
Total I	I1 + I2	#23	#21 AND #22	327
Reference test (R)				
Diagnosis of interest (D)	D1 Respiratory distress syndrome	#24	“Respiratory distress syndrome”	8,077
Total		#25	#18 AND #23 AND #24	30

Supplementary S3: Funnel plot asymmetry test for publication bias in diagnostic accuracy studies of fetal lung volume.

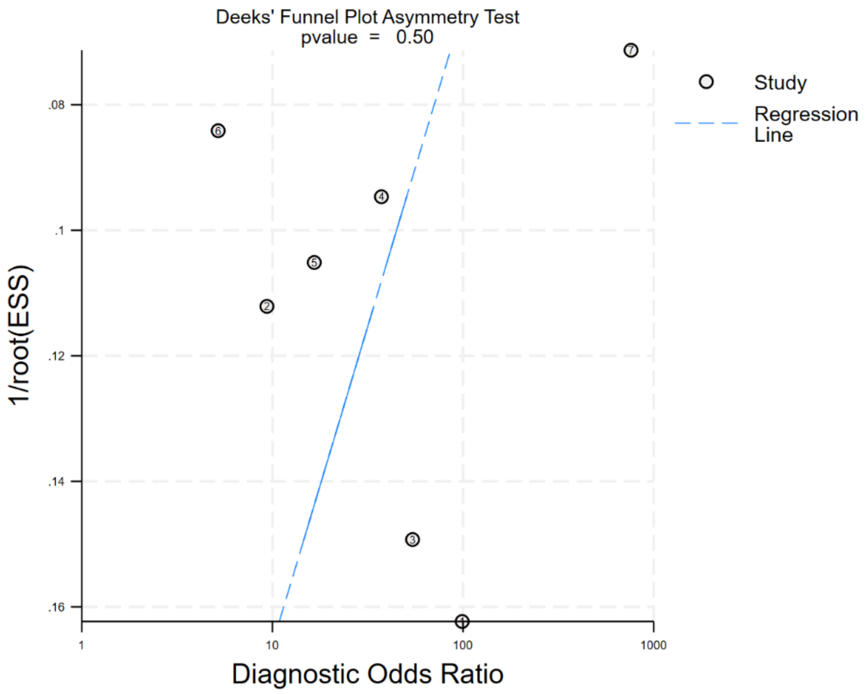

Deeks' Funnel Plot Asymmetry Test
pvalue = 0.50
1/root(ESS)
.08
.1
.12
.14
.16
1
10
100
1000
Diagnostic Odds Ratio
Study
Regression Line
